# ﻿Four new species of the genus *Andixius* Emeljanov & Hayashi (Hemiptera, Fulgoromorpha, Cixiidae) from China

**DOI:** 10.3897/zookeys.1141.84564

**Published:** 2023-01-19

**Authors:** Xiao-Ya Wang, Yan Zhi, Lin Yang, Xiang-Sheng Chen

**Affiliations:** 1 Institute of Entomology, Guizhou University, Guiyang, Guizhou 550025, China; 2 The Provincial Special Key Laboratory for Development and Utilization of Insect Resources of Guizhou, Guizhou University, Guiyang, Guizhou, 550025, China; 3 Laboratory Animal Center, Guizhou Medical University, Guiyang, Guizhou 550025, China

**Keywords:** Andini, Fulgoroidea, morphology, taxonomy

## Abstract

Four new species of the genus *Andixius* Emeljanov & Hayashi, 2007 are described and illustrated from China. These are *A.flagellihamus* Wang & Chen, **sp. nov.**, *A.gracilispinus* Wang & Chen, **sp. nov.**, *A.productus* Wang & Chen, **sp. nov.** and *A.truncatus* Wang & Chen, **sp. nov.** Photographs of the new species and an identification key to all *Andixius* species are provided.

## ﻿Introduction

The planthopper tribe Andini (Hemiptera, Auchenorrhyncha, Fulgoromorpha, Cixiidae, Cixiinae) consists of 129 species in three genera worldwide (Bourgoin 2022; Wang et al. 2022). Within the tribe Andini, *Andixius* is a small genus established by Emeljanov and Hayashi (2007) with two species, *A.nupta* Emeljanov & Hayashi, 2007 (as its type species) and *A.venustus* (Tsaur & Hsu, 1991) (previously placed in the genus *Brixia* Stål, 1856). Zhi et al. (2018) added two more species, *A.longispinus* and *A.trifurcus*, to the genus. Later, Wang et al. (2020) described two new species: *A.cultratus* and *A.lingulatus*.

Recent study of some Chinese specimens has found four new species, *A.flagellihamus* Wang & Chen, sp. nov., *A.gracilispinus* Wang & Chen, sp. nov., *A.productus* Wang & Chen, sp. nov. and *A.truncatus* Wang & Chen, sp. nov., which are described here. Hence, the number of *Andixius* species is now 10, with nine species occurring in China.

## ﻿Materials and methods

The morphological terminology follows Bourgoin (1987) and Bourgoin et al. (2015). The morphological terminology of female genitalia follows Bourgoin (1993). Dry specimens were used for the descriptions and illustrations. Body length was measured from the apex of the vertex to the tip of the forewing; vertex length was measured at the median length of the vertex (from the apical transverse carina to the tip of the basal emargination). Observations and drawings of external morphology were made with the aid of a Leica MZ 12.5 stereomicroscope. Photographs of the types were taken with the Keyence VHX-1000 system. Illustrations were scanned with a CanoScan LiDE 200 scanner and imported into Adobe Photoshop CS7 for labelling and plate composition. The dissected male genitalia are preserved in glycerine in small plastic tubes pinned together with the specimens.

The type specimens are deposited in the Institute of Entomology, Guizhou University, Guiyang, Guizhou Province, China (**IEGU**).

## ﻿Taxonomy

### ﻿ Andixius

Taxon classificationAnimaliaHemipteraCixiidae

Emeljanov & Hayashi, 2007

82C5BC63-F0EB-59CD-989A-7B59AFE2B2F4

Andixius Emeljanov & Hayashi, 2007: 127; Zhi et al. 2018: 56; Wang et al. 2020: 441.

#### Type species.

*Andixiusnupta* Emeljanov & Hayashi, 2007, original designation.

#### Diagnosis.

The distinctive characters proposed by Zhi et al. (2018) are modified as follows: head including eyes distinctly narrower than pronotum. Lateral carinae of frons and postclypeus foliate. Rostrum long, extended considerably beyond hind coxae. Forewings without trifid branching of ScP+R and MP near basal cell, ScP+R (ScP+RA and RP) forming a short common stalk. Legs simple, fore coxae without angular apical lobe, hind tibia with several small lateral spines.

#### Distribution.

China (Guangdong, Guangxi, Taiwan, Xizang, Yunnan), Japan (Ryukyu Islands).

##### ﻿Checklist and distributions of species of *Andixius* Emeljanov & Hayashi

*A.cultratus* Wang, Zhi & Chen, 2020; China (Guangdong).

*A.flagellihamus* Wang & Chen, sp. nov.; China (Xizang).

*A.gracilispinus* Wang & Chen, sp. nov.; China (Xizang).

*A.lingulatus* Wang, Zhi & Chen, 2020; China (Guangxi).

*A.longispinus* Zhi & Chen, 2018; China (Yunnan).

*A.nupta* Emeljanov & Hayashi, 2007; Japan (Ryukyus).

*A.productus* Wang & Chen, sp. nov.; China (Xizang).

*A.trifurcus* Zhi & Chen, 2018; China (Yunnan).

*A.truncatus* Wang & Chen, sp. nov.; (Guangxi).

*A.venustus* (Tsaur & Hsu, 1991); China (Taiwan).

### ﻿Key to species of *Andixius* (males) Emeljanov & Hayashi

**Table:** 

1	Anal segment symmetrical dorsally	**2**
–	Anal segment asymmetrical dorsally	**7**
2	Apical right side of periandrium with a large linguiform laminal process (Wang et al. 2020: figs 31–34)	***A.lingulatus***
–	Apical right side of periandrium without linguiform laminal process	**3**
3	Ventral margin of periandrium with a projection, of which basal 1/3 longitudinally and 2/3 horizontally extended, endosoma with two simple processes, not bifurcate (Zhi et al. 2018: figs 13–16)	***A.longispinus***
–	Periandrium without above spinose process	**4**
4	Periandrium with an expanded semi-enclosed structure around the left side and ventral margin of periandrium (Zhi et al. 2018: figs 25–28)	***A.trifurcus***
–	Periandrium without expanded semi-enclosed structure	**5**
5	Left side of periandrium with a bifurcate process (Emeljanov and Hayashi 2007: figs 11–13)	***A.nupta***
–	Left side of periandrium without process or process on left side of periandrium not bifurcated	**6**
6	Dorsal margin of endosoma with a large spinose process (Wang et al. 2020: figs 10–13)	***A.cultratus***
–	Right side of endosoma with a bifurcated production (Tsaur et al. 1991: fig. 33D–F)	***A.venustus***
7	Endosoma of aedeagus with a hooked spinose process apically (Fig. 3F)	***A.flagellihamus* sp. nov.**
–	Endosoma of aedeagus without above spinose process	**8**
8	Middle dorsal margin of periandrium with a slightly stout and long spinose process (Fig. 7E–H)	***A.productus* sp. nov.**
–	Middle dorsal margin of periandrium without spinose process	**9**
9	Left apical side of ventral margin of periandrium with a triangular laminal process, of which middle right side concaved heavily, forming two large processes (Fig. 5F)	***A.gracilispinus* sp. nov.**
–	Ventral margin of periandrium with a broad laminal process, apex slightly truncate (Fig. 10E, F)	***A.truncatus* sp. nov.**

### ﻿ Andixius flagellihamus

Taxon classificationAnimaliaHemipteraCixiidae

Wang & Chen sp. nov.

4A130AF2-7552-5EC5-BFB7-D1E5DCAE7E08

https://zoobank.org/5BB7E534-9C4C-4507-8FA5-CA5C1D3D5646

**Figure 1.**
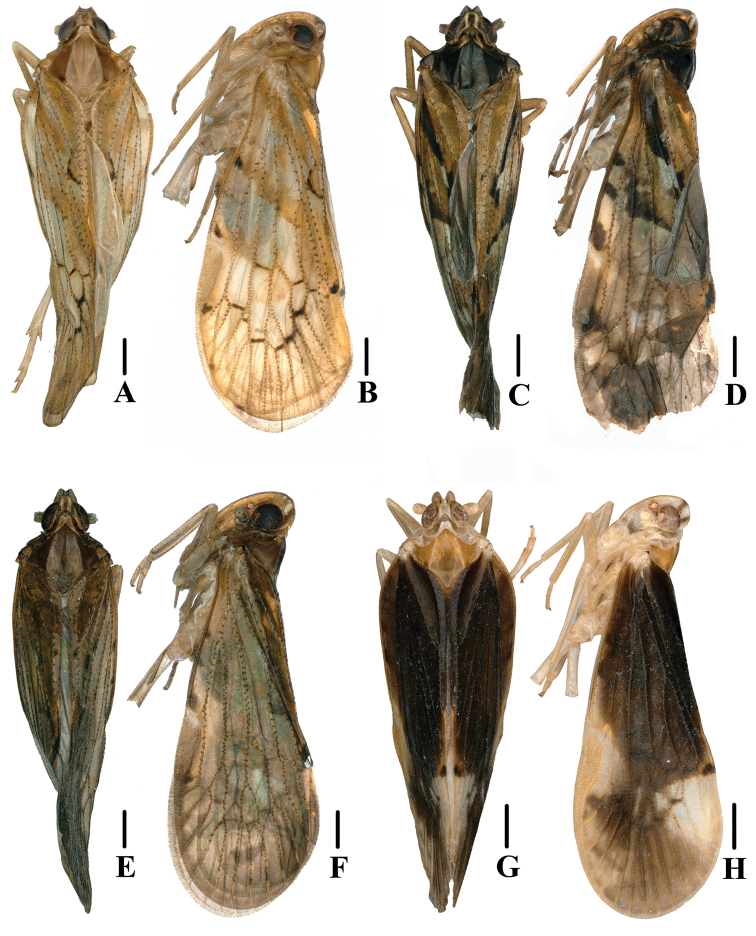
New species of *Andixius***A, B***A.flagellihamus* sp. nov., male **A** dorsal view **B** lateral view **C, D***A.gracilispinus* sp. nov., male **C** dorsal view **D** lateral view **E, F***A.productus* sp. nov., male **E** dorsal view **F** lateral view **G, H***A.truncatus* sp. nov., male **G** dorsal view **H** lateral view. Scale bars: 0.5 mm.

**Figure 2.**
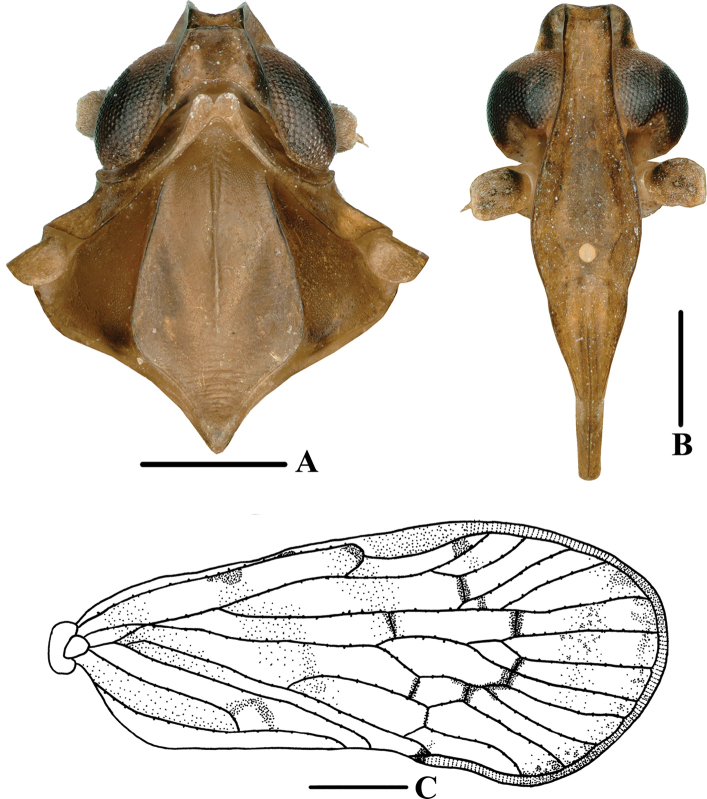
*Andixiusflagellihamus* sp. nov., male **A** head and thorax, dorsal view **B** face, ventral view **C** forewing. Scale bars: 0.5 mm (**A, B**); 1.0 mm (**C**).

**Figure 3.**
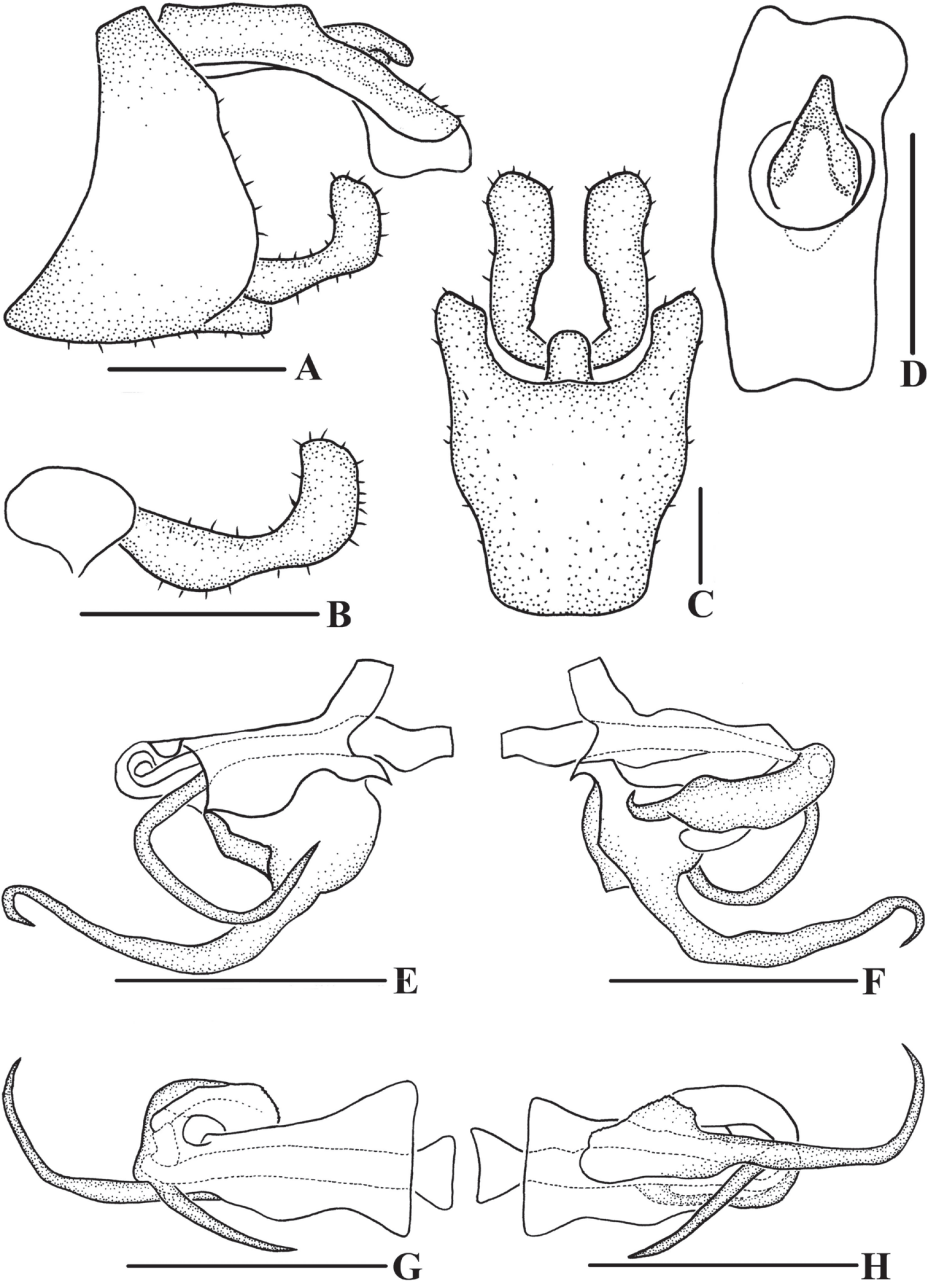
*Andixiusflagellihamus* sp. nov., male **A** genitalia, lateral view **B** gonostyli, lateral view **C** pygofer and gonostyli, ventral view **D** anal segment, dorsal view **E** aedeagus, right side **F** aedeagus, left side **G** aedeagus, dorsal view **H** aedeagus, ventral view. Scale bars: 0.5 mm.

Figs 1A, B , 2A–C , 3A–H

#### Type material.

***Holotype***: ♂, **China**: Xizang Province, Medog County, Beibeng Town (29.2483°N, 95.1819°E), 15 August 2020, Yongjin Sui leg.; ***Paratypes***: ♂, same data as holotype.

#### Description.

Body length: male 6.65–7.00 mm (*n* = 2).

***Coloration*.** General color light yellowish brown (Figs 1A, B, 2A, B). Eyes black-brown, ocelli faint yellowish brown, semitranslucent. Lateral margin of frons yellowish brown, behind eyes with two brown spots. Antenna, vertex, face, and rostrum generally yellowish brown. Pronotum and mesonotum yellowish brown. Forewing semitranslucent, with veins and stigma yellowish brown, tubercles black-brown; costal vein, slightly in front of and behind stigma and near claval fork with an irregular puce spot. Hind tibiae yellowish brown. Ventral abdomen brown.

***Head and thorax*.** Vertex (Figs 1A, 2A) 1.14 times longer than wide; anterior and posterior margin slightly recessed, lateral carinae developed, median carina absent. Frons (Fig. 2B) claviform, 2.58 times as long as wide. Pronotum (Figs 1A, 2A) as long as vertex. Mesonotum 1.40 times longer than pronotum and vertex combined, lateral carinae curved outwards. Forewing (Figs 1B, 2C) 2.28 times longer than wide, with 12 apical cells and six subapical cells; RP 3 branches, MP with five terminals: MP_11_, MP_12_, MP_2_, MP_3_, and MP_4_, fork MP_1_+MP_2_ basad of fork MP_3_+MP_4_. Hind tibia with three lateral spines; chaetotaxy of hind tarsi 6/7.

***Male genitalia*.** Pygofer (Fig. 3A, C) symmetrical. Medioventral process rounded protruding in ventral view. Anal segment (Fig. 3A, D) asymmetrical, apical margin expanded downwards in lateral view; 2.28 times longer than wide in dorsal view; anal style strap-shaped, not beyond anal segment. Gonostyli (Fig. 3B, C) symmetrical ventrally; in inner lateral view, base slender, apex enlarged. Aedeagus (Fig. 3E–H) with five processes. In left side view, basal ventral margin of periandrium protruding; in right side view, base of periandrium with a U-shaped spinose process, directed cephalad; in ventral view, near base of periandrium with a long spinose process, apex curved upwards, forming a hooked process, directed cephalad. Endosoma somewhat long, apex with a hooked spinose process.

#### Distribution.

China (Xizang) (Fig. 12).

#### Etymology.

The specific name is derived from the Latin adjective *flagellihamus*, referring to the 1-hooked spinose process arising from the apex of the endosoma.

#### Remarks.

This species can be distinguished from the other species of the genus by the following characters: basal right side of periandrium with a U-shaped spinose process; basal ventral margin of periandrium with a long spinose process, apex curved upwards, forming a hooked process; apex of endosoma with a hooked spinose process.

### ﻿ Andixius gracilispinus

Taxon classificationAnimaliaHemipteraCixiidae

Wang & Chen sp. nov.

BECD8207-9CC0-51A5-91D1-51B9D3F7165C

https://zoobank.org/4CF579B2-3F5B-4926-99D8-59E7F8E16E85

**Figure 4.**
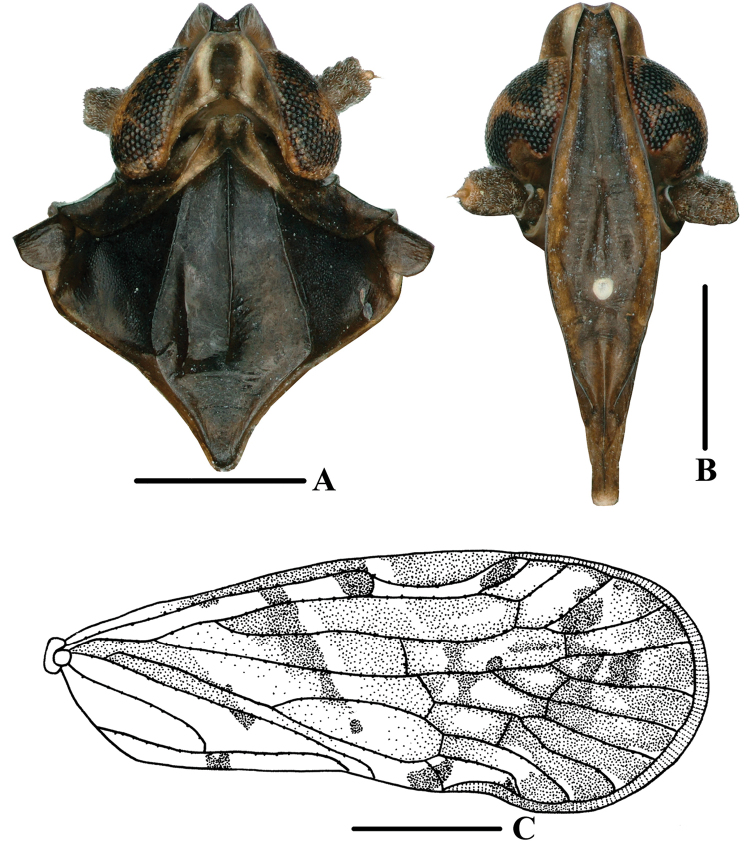
*Andixiusgracilispinus* sp. nov., male **A** head and thorax, dorsal view **B** face, ventral view **C** forewing. Scale bars: 0.5 mm (**A, B**); 1.0 mm (**C**).

**Figure 5.**
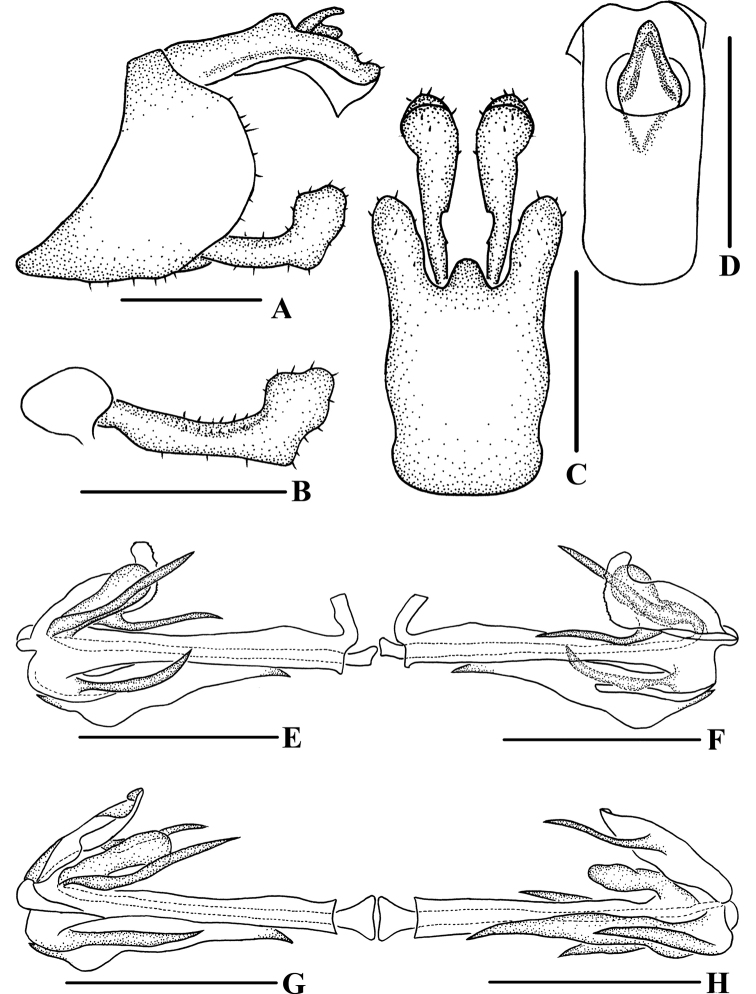
*Andixiusgracilispinus* sp. nov., male **A** genitalia, lateral view **B** gonostyli, lateral view **C** pygofer and gonostyli, ventral view **D** anal segment, dorsal view **E** aedeagus, right side **F** aedeagus, left side **G** aedeagus, dorsal view **H** aedeagus, ventral view. Scale bars: 0.5 mm.

Figs 1C, D , 4A–C , 5A–H

#### Type material.

***Holotype***: ♂, **China**: Xizang Province, Bomê County, Yigong Town, Tongmai Village (30.1071°N, 95.0867°E), 18–20 August 2020, Yongjin Sui leg.; ***Paratypes***: ♂, same data as holotype.

#### Description.

Body length: male 5.63–5.82 mm (*n* = 2).

***Coloration*.** General color yellowish brown (Figs 1C, D, 4A, B). Eyes black-brown, ocelli faint light yellowish brown, semitranslucent. Lateral margin of frons yellowish brown, behind eyes with an off-white spot. Antenna and vertex yellowish brown. Face and rostrum dark fawn. Pronotum and mesonotum black. Forewing semitranslucent, with veins, stigma, and tubercles black-brown; basal and middle part of forewings with an inner oblique stripe; base and lateral margin black-brown; in front of fork CuA_1_+CuA_2_ with a pale spot; costal vein with three small, spaced, dark brown spots; behind stigma and near claval fork with an irregular puce spot; apical half of wing with brown patches. Hind tibiae light brown. Ventral abdomen brown.

***Head and thorax*.** Vertex (Figs 1C, 4A) 1.37 times longer than wide; anterior margin slightly curved, recessed; posterior margin V-shaped, recessed; lateral carinae developed; median carina absent. Frons (Fig. 4 B) claviform, 2.85 times as long as wide. Pronotum (Figs 1C, 4A) slightly shorter than vertex. Mesonotum 1.34 times longer than pronotum and vertex combined, lateral carinae curved outwards. Forewing (Figs 1D, 4C) 2.57 times longer than wide, with 11 apical cells and six subapical cells; RP 3 branches, MP with five terminals: MP_11_, MP_12_, MP_2_, MP_3_, and MP_4_, fork MP_1_+MP_2_ basad of fork MP_3_+MP_4_. Hind tibia with five lateral spines; chaetotaxy of hind tarsi 6/6.

***Male genitalia*.** Pygofer (Fig. 5A, C) symmetrical. Medioventral process rounded protruding in ventral view. Anal segment (Fig. 5A, D) asymmetrical, left lobe larger than right lobe, dorsal margin almost straight, apical margin slightly expanded downwards in lateral view; 2.44 times longer than wide in dorsal view; anal style strap-shaped, not beyond anal segment. Gonostyli (Fig. 5B, C) ventrally symmetrical; in inner lateral view, middle part slender but base and apex enlarged. Aedeagus (Fig. 5E–H) with three processes. In left side view, basal ventral margin of periandrium with a triangular laminal process, of which middle right side concaved heavily, forming two large processes, one directed cephalad, another directed caudad, basal dorsal margin of periandrium with a laminal process, of which near apex of dorsal margin recessed, apex convex, left side of margin dentate; in right side view, apical ventral margin of periandrium projecting, near apex with a long spinose process, curved upwards, directed dorsocephalad; in dorsal view, laminal process grooved, arising at left side of basal dorsal margin of periandrium, left side convex, apical right side rolling, middle part concave in a right angle, apex of periandrium with a long spinose process, slightly curved, directed cephalad; in ventral view, near apex of grooved laminal process with a long spinose process, slightly curved, directed cephalad. Endosoma slightly sclerotized, without process.

#### Distribution.

China (Xizang) (Fig. 12).

#### Etymology.

The specific name is derived from the Latin adjective *gracilispinus*, referring to the one long spinose process arising from the apical right side of the ventral margin of the periandrium.

#### Remarks.

Male genitalia of *A.gracilispinus* sp. nov. are similar to *A.venustus* Tsaur & Hsu, 1991 in appearance, but differs in: (1) basal left side of ventral margin of periandrium with a triangular laminal process, of which middle right side concaved heavily, forming two large processes (*A.venustus* with a spinose process in the same position); (2) near apical right side of ventral margin of periandrium with a long spinose process, slightly curved (right side of ventral margin of periandrium without spinose process in *A.venustus*); (3) basal dorsal margin of periandrium with a grooved laminal process (without process in *A.venustus*).

### ﻿ Andixius productus

Taxon classificationAnimaliaHemipteraCixiidae

Wang & Chen sp. nov.

F58ABCA6-C31E-5A1D-A365-33C0937A010C

https://zoobank.org/AE3FAE97-597C-41D8-92B8-7C8A2CCB5464

**Figure 6.**
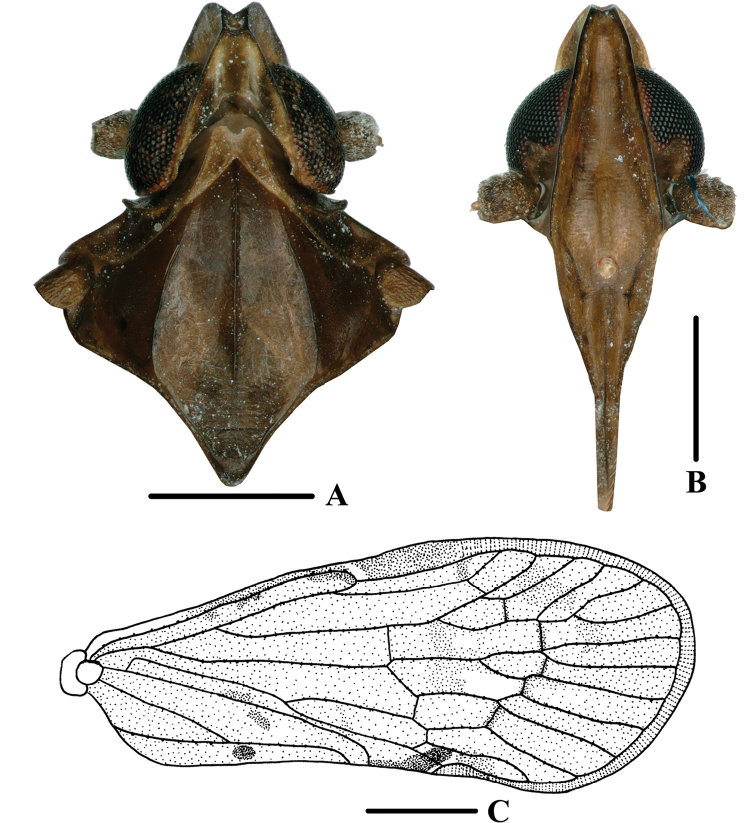
*Andixiusproductus* sp. nov., male **A** head and thorax, dorsal view **B** face, ventral view **C** forewing. Scale bars: 0.5 mm (**A, B**); 1.0 mm (**C**).

**Figure 7.**
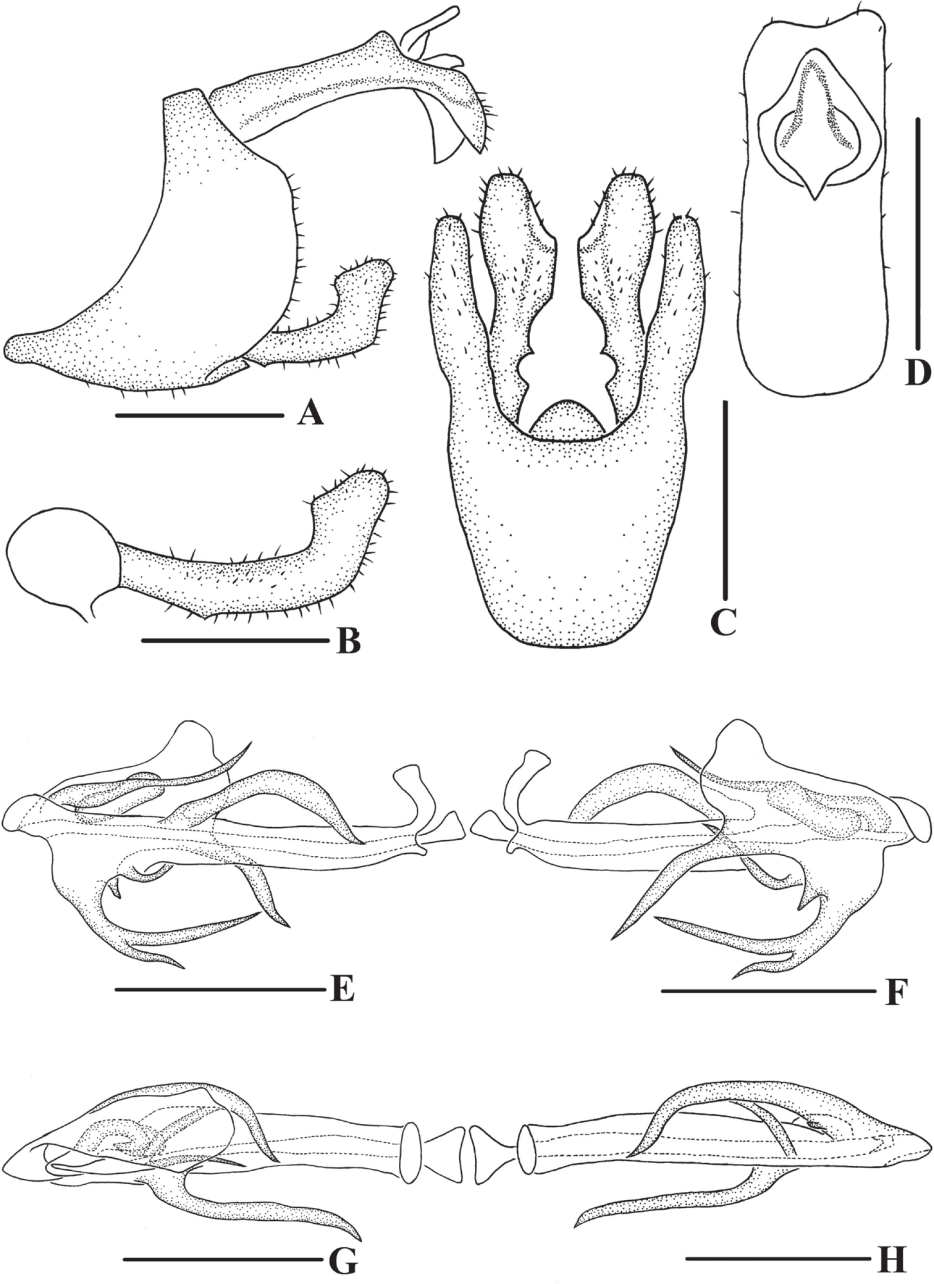
*Andixiusproductus* sp. nov., male **A** genitalia, lateral view **B** gonostyli, lateral view **C** pygofer and gonostyli, ventral view **D** anal segment, dorsal view **E** aedeagus, right side **F** aedeagus, left side **G** aedeagus, dorsal view **H** aedeagus, ventral view. Scale bars: 0.5 mm.

**Figure 8.**
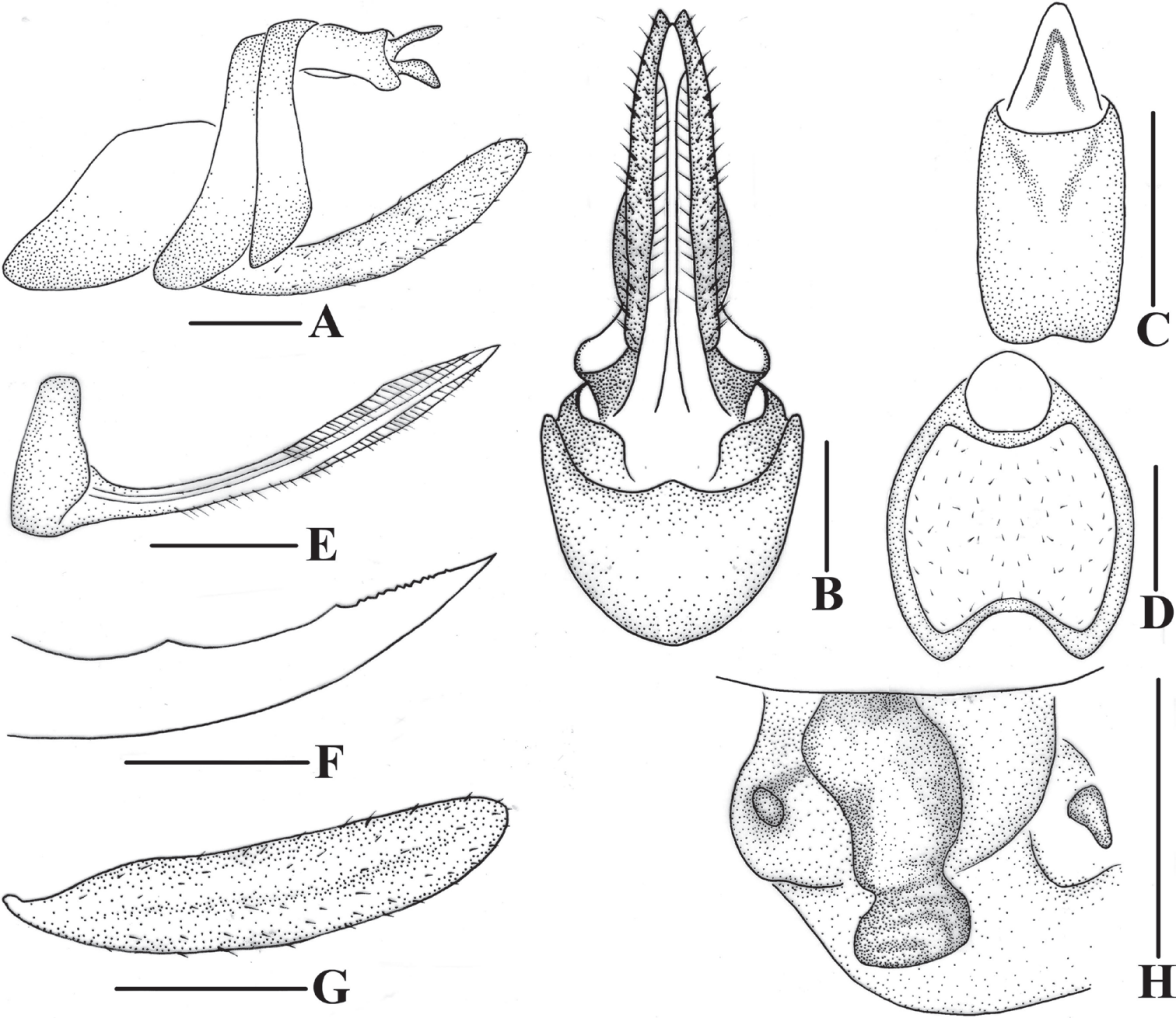
*Andixiusproductus* sp. nov., female **A** genitalia, lateral view **B** genitalia, ventral view **C** anal segment, dorsal view **D** tergite IX, caudal view **E** gonapophysis VIII and gonocoxa VIII, dorsal view **F** gonapophysis IX, lateral view **G** gonoplac, inner lateral view **H** posterior vagina, ventral view. Scale bars: 0.5 mm.

Figs 1E, F , 6A–C , 7A–H , 8A–H

#### Type material.

***Holotype***: ♂, **China**: Xizang Province, Medog County, Damu Town, 80K (29.6237°N, 95.4888°E), 18 August 2020, Yongjin Sui leg.; ***Paratypes***: 6♂♂ 2♀♀, same data as holotype.

#### Description.

Body length: male 5.71–6.90 mm (*n* = 7), female 7.78–7.90 mm (*n* = 2).

***Coloration*.** General color black-brown (Figs 1E, F, 6A, B). Eyes black-brown, ocelli faint yellowish brown, semitranslucent. Lateral margin of frons yellowish brown, behind eyes with an off-white spot. Antenna and vertex black-brown. Face and rostrum dark fawn. Pronotum and mesonotum black-brown. Forewing semitranslucent, generally black-brown, veins and stigma yellowish brown, tubercles black-brown; costal vein, in the middle of, behind and near claval fork with deep-brown spots. Hind tibiae light brown. Ventral abdomen brown.

***Head and thorax*.** Vertex (Figs 1E, 6A) 1.65 times longer than wide; anterior margin slightly curved, recessed; posterior margin V-shaped, recessed; lateral carinae developed; median carina absent. Frons (Fig. 6B) claviform, 2.90 times as long as wide. Pronotum (Figs 1E, 6A) as long as vertex. Mesonotum 1.21 times longer than pronotum and vertex combined, lateral carinae curved outwards. Forewing (Figs 1F, 6C) 2.49 times longer than wide, with 13 apical cells and seven subapical cells; RP 3 branches, MP with five terminals: MP_11_, MP_12_, MP_2_, MP_3_, and MP_4_, fork MP_1_+MP_2_ basad of fork MP_3_+MP_4_. Hind tibia with five lateral spines; chaetotaxy of hind tarsi 6/5.

***Male genitalia*.** Pygofer (Fig. 7A, C) symmetrical. Medioventral process rounded protruding in ventral view. Anal segment (Fig. 7A, D) long, tubular, with dorsal margin almost straight and apical margin slightly expanded downwards in lateral view; 2.67 times longer than wide in dorsal view; anal style strap-shaped, not beyond anal segment. Gonostyli (Fig. 7B, C) symmetrical ventrally, inner margin with a small spinose process near base, apex enlarged; in lateral view, near apex bending upwards. Aedeagus (Fig. 7E–H) with seven processes. In left side view, periandrium with an expanded laminal process around the left side and dorsal margin of periandrium; ventral margin of the expanded structure with two spinose processes, upper one slender, slightly curved, directed ventrocephalad, lower one small, directed ventrad; in right side view, apical ventral margin of periandrium projecting, the process expanded downwards, apex bifurcated, forming two spinose processes, the dorsal one long, another short, directed cephalad, the right side of the process with a long spinose process, bending around the periandrium, directed left-dorsocephalad, apical dorsal margin of periandrium with a long spinose process, near apex slightly curved upwards, directed dorsocephalad; in dorsal view, laminal process covering dorsal margin, middle part of periandrium with a thick, long spinose process, slightly curved, directed cephalad. Endosoma short, slightly sclerotized, without process.

***Female genitalia*.** Tergite IX (Fig. 8A, B, D) moderately sclerotized, with a large nearly elliptical wax plate. Anal segment (Fig. 8C) rectangular, 2.43 times longer than wide in dorsal view, anal style linguiform. Gonapophysis IX (Fig. 8F) with one middle tooth; distance ratio between middle tooth to apex and length of denticulate portion is 1.98. Gonoplac (Fig. 8G) rod-like, 4.44 times longer than wide in lateral view. Posterior vagina pattern as shown in Fig. 8H.

#### Distribution.

China (Xizang) (Fig. 12).

#### Etymology.

The specific name is derived from the Latin adjective *productus*, referring to the one long spinose process arising from the apical ventral margin of the periandrium.

#### Remarks.

Male genitalia of *A.productus* sp. nov. are similar to *A.trifurcus* Zhi & Chen, 2018, but differs in: (1) periandrium with an expanded laminal process around the left side and dorsal margin of periandrium (laminal process around the left side, dorsal margin and ventral margin in *A.trifurcus*); (2) basal ventral margin of periandrium with laminal process, of which ventral margin with two processes (*A.trifurcus* with three long spinose processes in the same position); (3) right side of ventral margin of periandrium with three spinose processes (right side of middle part of periandrium with a spinose process in *A.trifurcus*); (4) middle part of periandrium with a thick and long spinose process (without process in *A.trifurcus*).

### ﻿ Andixius truncatus

Taxon classificationAnimaliaHemipteraCixiidae

Wang & Chen sp. nov.

6AAFC595-BABB-5FB0-862A-EE78D8C83C99

https://zoobank.org/1EE35403-E65B-43C5-B171-54C7CC313B23

**Figure 9.**
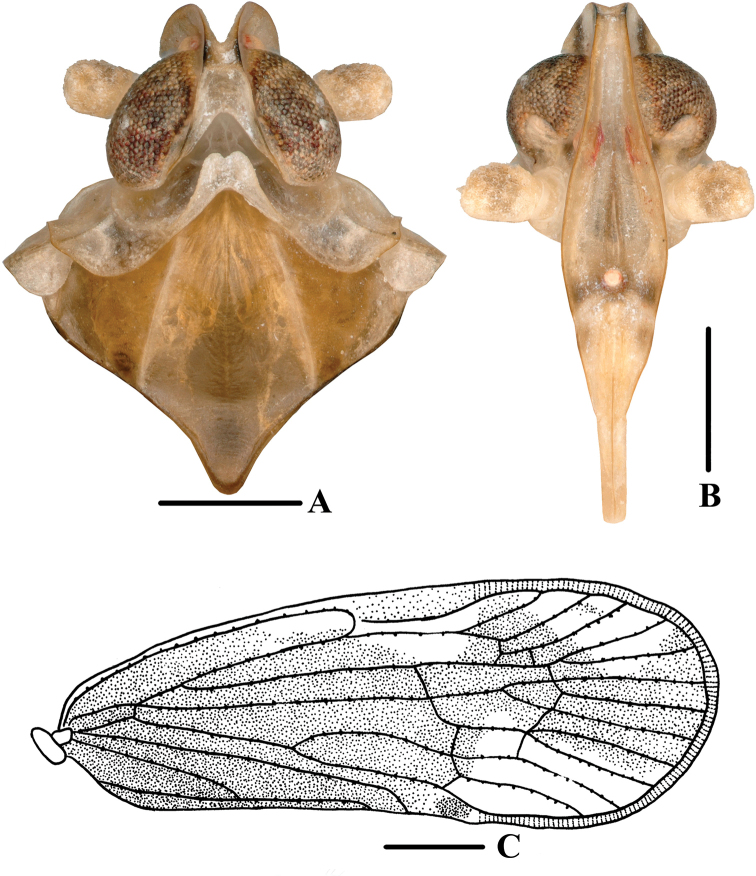
*Andixiustruncatus* sp. nov., male **A** head and thorax, dorsal view **B** face, ventral view **C** forewing. Scale bars: 0.5 mm (**A, B**); 1.0 mm (**C**).

**Figure 10.**
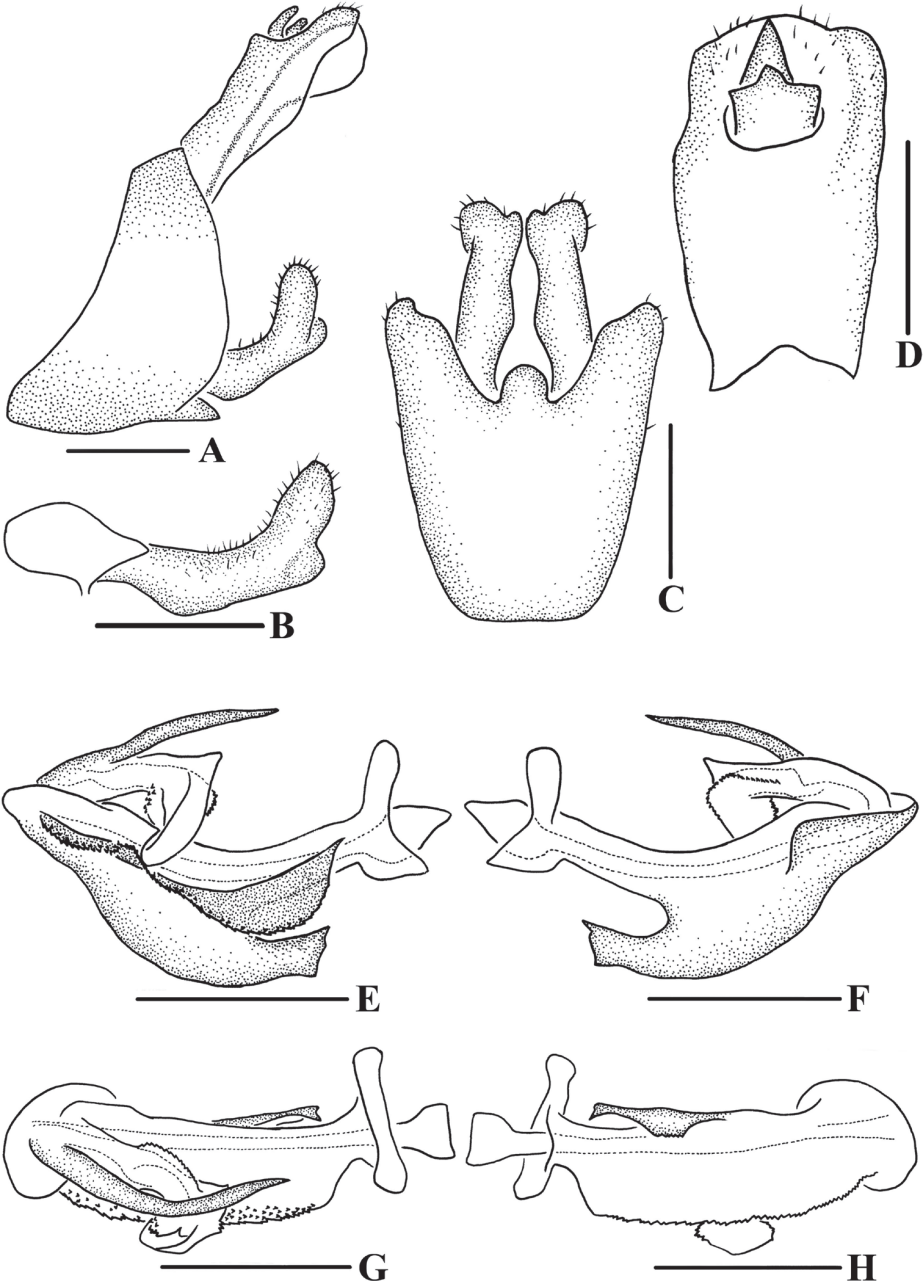
*Andixiustruncatus* sp. nov., male **A** genitalia, lateral view **B** gonostyli, lateral view **C** pygofer and gonostyli, ventral view **D** anal segment, dorsal view **E** aedeagus, right side **F** aedeagus, left side **G** aedeagus, dorsal view **H** aedeagus, ventral view. Scale bars: 0.5 mm.

**Figure 11.**
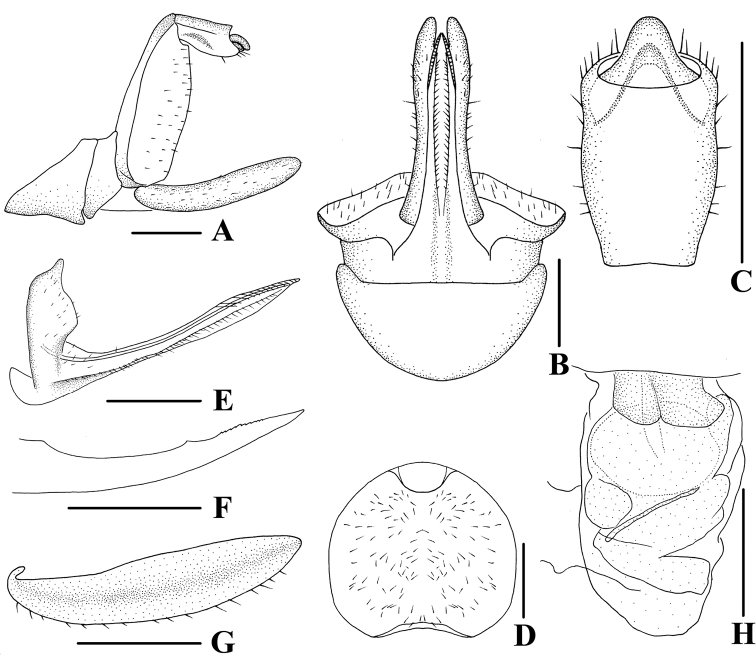
*Andixiustruncatus* sp. nov., female **A** genitalia, lateral view **B** genitalia, ventral view **C** anal segment, dorsal view **D** tergite IX, caudal view **E** gonapophysis VIII and gonocoxa VIII, dorsal view **F** gonapophysis IX, lateral view **G** gonoplac, inner lateral view **H** posterior vagina, ventral view. Scale bars: 0.5 mm.

Figs 1G, H , 9A–C , 10A–H , 11A–H

#### Type material.

***Holotype***: ♂, **China**: Guangxi Province, Longsheng County, Huaping National Natural Reserve (25.6046°N, 109.9417°E), 18 July 2020, Xiaoya Wang, Yongjin Sui, Zhicheng Zhou and Jing Wang leg.; ***Paratypes***: 9♂♂ 5♀♀, same data as holotype.

#### Description.

Body length: male 6.56–7.20 mm (*n* = 10), female 7.25–8.86 mm (*n* = 5).

***Coloration*.** General color black-brown (Figs 1G, H, 9A, B). Eyes yellowish brown, ocelli faintly yellow, semitranslucent. Lateral margin of frons yellowish white, behind eyes with two brown spots. Antenna, vertex, face, and rostrum fawn. Pronotum fawn. Mesonotum yellowish brown. Forewing semitranslucent, generally black-brown; stigma fawn; veins and tubercles the same color as the wing surface; slightly below stigma and near claval fork with an irregular, yellowish-white spot; apical half of wing light brown. Hind tibiae yellowish brown. Ventral abdomen yellowish brown.

***Head and thorax*.** Vertex (Figs 1G, 9A) 2.76 times longer than wide; anterior margin slightly curved recessed, posterior margin U-shaped, recessed; lateral carinae developed; median carina absent. Frons (Fig. 9B) claviform, 3.00 times as long as wide. Pronotum (Figs 1G, 9A) slightly shorter than vertex. Mesonotum 1.08 times slightly longer than pronotum and vertex combined, lateral carinae curved outwards. Forewing (Figs 1H, 9C) 2.74 times longer than wide, with 12 apical cells and seven subapical cells; RP 3 branches, MP with five terminals: MP_11_, MP_12_, MP_2_, MP_3_, and MP_4_, fork MP_1_+MP_2_ basad of fork MP_3_+MP_4_. Hind tibia with four lateral spines; chaetotaxy of hind tarsi 8/8.

***Male genitalia*.** Pygofer (Fig. 10A, C) symmetrical. Medioventral process rounded protruding in ventral view. Anal segment (Fig. 10A, D) tubular, dorsal margin almost straight, ventral margin slightly curved, right lobe larger than left lobe in lateral view; 1.81 times longer than wide in dorsal view; anal style strap-shaped, not beyond anal segment. Gonostyli (Fig. 10B, C) symmetrical ventrally, inner margin with a small process near base; in lateral view, apex enlarged and foot-shaped. Aedeagus (Fig. 10E–H) with three processes. In left side view, apex of periandrium with laminal process, apex rounded; in right side view, ventral margin of periandrium with a long, broad laminal process, apex truncated, margin with small teeth, directed cephalad, with endosoma curving ventrally at a right angle; in dorsal view, base of endosoma with a long spinose process, slightly curved, directed dorsocephalad.

***Female genitalia*.** Tergite IX (Fig. 11A, B, D) moderately sclerotized, with a large, nearly circular wax plate. Anal segment (Fig. 11C) rectangular, 1.60 times longer than wide in dorsal view, anal style linguiform. Gonapophysis IX (Fig. 11F) with one middle tooth; distance ratio between middle tooth to apex and length of denticulate portion is 2.70. Gonoplac (Fig. 11G) rod-like, 4.3 times longer than wide in lateral view. Posterior vagina pattern as shown in Fig. 11H.

#### Distribution.

**Figure 12.**
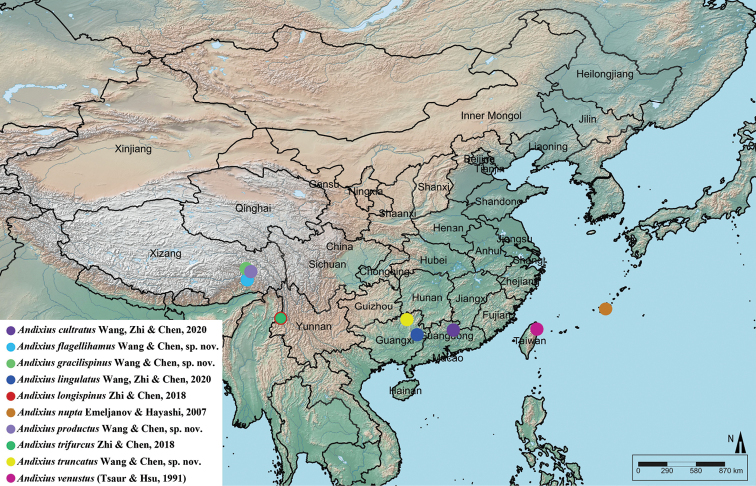
Geographic distribution of *Andixius* species.

China (Guangxi) (Fig. 12).

#### Etymology.

The specific name is derived from the Latin adjective *truncatus*, referring to the ventral margin of periandrium with a long, broad laminal process having a truncated apex.

#### Remarks.

This species can be distinguished from the other *Andixius* species by the following characters: forewing general black-brown, with an irregular yellowish-white spot slightly below stigma and near claval fork; ventral margin of periandrium with a long and broad laminal process, apex truncated, margin with small teeth; endosoma curving ventrally in right angle, base of dorsal margin with a long spinose process.

## ﻿Discussion

The present discovery of four new species in the genus *Andixius* once again emphasizes the need for further study on the group based on male genitalia whenever possible (Zhi et al. 2018; Wang et al. 2020). Five species of this genus were previously described from southern China. With expanded collection efforts, our team went to Xizang Province in southwestern China, where it had not been, and there we found three of the new species described in the paper. Xizang Province has a high altitude, but it is rich in species and productive for making collections. Additionally, we found a new species with distinctive coloration in Guangxi Province.

Nine *Andixius* species are now known to occur in China, which can be certainly considered to be an underestimate, as the fauna is far from being well known in this interesting region. Therefore, further investigation should be considered to fill the faunistic gaps, as it is obvious that many more taxa remain to be discovered and described.

Currently the tribe Andini includes 129 species in three genera (*Parandes* Muir, 1925, *Andes* Stål, 1866, and *Andixius*), of which only the latter two genera and 18 species occur in China (Bourgoin 2022; Wang et al. 2022). A comparison of *Andes*, *Andixius*, and *Parandes* shows that species in these genera look rather similar, but these genera can be easily distinguished by the veins and fore coxa. The forewings of *Andixius* are without trifid branching of ScP+R and MP near the basal cell, and ScP+R (ScP+RA and RP) forming a short common stalk, while ScP, RP and MP emerge independently or very close to the basal cell in the other Andini genera. The outer edge of the apical half of the fore coxae is extended and smoothly protruding in *Parandes*, but the outer edge of the apical half of the fore coxae is straight and does not extend in *Andes*.

## Supplementary Material

XML Treatment for Andixius

XML Treatment for Andixius flagellihamus

XML Treatment for Andixius gracilispinus

XML Treatment for Andixius productus

XML Treatment for Andixius truncatus
